# Clinician experiences of healthy lifestyle promotion and perceptions of digital interventions as complementary tools for lifestyle behavior change in primary care

**DOI:** 10.1186/s12875-018-0829-z

**Published:** 2018-08-21

**Authors:** Anne H. Berman, Karoline Kolaas, Elisabeth Petersén, Preben Bendtsen, Erik Hedman, Catharina Linderoth, Ulrika Müssener, Kristina Sinadinovic, Fredrik Spak, Ida Gremyr, Anna Thurang

**Affiliations:** 1 Center for Psychiatry Research, Department of Clinical Neuroscience, Karolinska Institutet & Stockholm Healthcare Services, Stockholm County Council, Norra Stationsgatan 69, SE-11364 Stockholm, Sweden; 2 Stockholm Center for Dependency Disorders, Box 17914, 118 95 Stockholm, Sweden; 3 Gustavsberg Primary Care Clinic, Odelbergs väg 19, Gustavsberg, Sweden; 4 Department of Medicine and Health Sciences, Linköping University, Linköping, Sweden; 5 Chalmers Technological University, Gothenburg, Sweden; 6 Department of Public Health and Community Medicine, Section for Epidemiology and Social Medicine, Sahlgrenska Academy, University of Gothenburg, Gothenburg, Sweden

**Keywords:** Clinician experiences, Digital interventions, E-health, Healthy lifestyle promotion, Primary care, Phenomenological hermeneutics, Qualitative research

## Abstract

**Background:**

Evidence-based practice for healthy lifestyle promotion in primary health care is supported internationally by national policies and guidelines but implementation in routine primary health care has been slow. Referral to digital interventions could lead to a larger proportion of patients accessing structured interventions for healthy lifestyle promotion, but such referral might have unknown implications for clinicians with patients accessing such interventions. This qualitative study aimed to explore the perceptions of clinicians in primary care on healthy lifestyle promotion with or without digital screening and intervention.

**Methods:**

Focus group interviews were conducted at 10 primary care clinics in Sweden with clinicians from different health professions. Transcribed interviews were analyzed using content analysis, with inspiration from a phenomenological-hermeneutic method involving naïve understanding, structural analysis and comprehensive understanding.

**Results:**

Two major themes captured clinicians’ perceptions on healthy lifestyle promotion: 1) the need for structured professional practice and 2) deficient professional practice as a hinder for implementation. Sub-themes in theme 1 were *striving towards professionalism,* which for participants meant working in a standardized fashion*,* with replicable routines regardless of clinic, as well as being able to monitor statistics on individual patient and group levels; and *embracing the future with critical optimism,* meaning expecting to develop professionally but also being concerned about the consequences of integrating digital tools into primary care, particularly regarding the importance of personal interaction between patient and provider. For theme 2, sub-themes were *being in an unmanageable situation*, meaning not being able to do what is perceived as best for the patient due to lack of time and resources; and *following one’s perception*, meaning working from a gut feeling, which for our participants also meant deviating from clinical routines.

**Conclusions:**

In efforts to increase evidence-based practice and lighten the burden of clinicians in primary care, decision- and policy-makers planning the introduction of digital tools for healthy lifestyle promotion will need to explicitly define their role as *complements* to face-to-face encounters. Our overriding hope is that this study will contribute to maintaining meaningfulness in the patient-clinician encounter, when digital tools are added to facilitate patient behavior change of unhealthy lifestyle behaviors.

**Electronic supplementary material:**

The online version of this article (10.1186/s12875-018-0829-z) contains supplementary material, which is available to authorized users.

## Background

Lifestyle-related illnesses and disease such as coronary heart disease, type 2 diabetes, and certain cancer forms have been leading causes of death worldwide over the past 25 years [1]. Healthy lifestyle promotion in primary health care has been supported internationally by national policies and guidelines based on evidence for the effectiveness of promotion of healthy lifestyle [2]. Despite the evidence for the effectiveness of promotion of health lifestyle interventions in primary health care, implementation in routine primary health care has been slow [3]. Many reasons have been put forward for the slow implementation of interventions for healthy lifestyles, not the least that it is time consuming for the individual clinician. This study addresses the potential of digital interventions as a means of facilitating increased healthy lifestyle promotion by clinicians in primary care.

In recent years a number of studies have shown the effectiveness of digital health behavior interventions, indicating that their effects could be equivalent to face-to-face interventions [4, 5]. Referral to digital interventions could lead to a larger proportion of patients accessing structured interventions for promoting a healthy lifestyle (e.g., [6, 7]). Referral to digital interventions might in turn require specific guidance from clinicians to patients accessing the interventions. An increasing amount of literature exists on the feasibility and effectiveness of guided or clinician-facilitated access to digital interventions for anxiety and depression [8–10]; there are fewer studies on facilitated access to interventions for healthy lifestyle promotion [11, 12]. Research on referral of patients by clinicians to digital applications promoting healthy lifestyle is still in its infancy [7, 12–15]. The lifestyle behavior that has been the focus of a large proportion of the research cited above on the effectiveness of digital interventions has concerned the promotion of sensible alcohol habits [11, 13, 14, 16, 17].

Implementing routine referral to digital health behavior intervention by clinicians in primary care may be a complex procedure and many questions are still unanswered. For example, how do clinicians currently support patients to promote a healthy lifestyle? To what extent are clinicians satisfied with their current way of working with lifestyle behaviors? How would they want to change their current methods, if at all? To what extent do clinicians use digital interventions today? Given a future scenario with systematic referral to digital interventions, what would be the advantages and disadvantages envisioned? Our preconception is that referral to digital interventions would certainly present challenges to routine procedures practiced by most clinicians in today’s primary health care context.

Digital interventions refer to the provision of treatment content to patients via digital tools, where the clinician’s role can vary widely. When the digital tools offer self-help, the clinician may not be involved at all, but self-help digital tools can be offered by the clinician through so-called facilitated access. Another variation is when the clinician offers direct guidance to the patient within a digital platform offering patient-clinician communication via secure messaging, chat or via telephone or video. The addition of digital interventions to the healthcare context falls under the wider concept of “e-health”, defined early on as a field “in the intersection of medical informatics, public health and business, referring to health services and information delivered or enhanced through the Internet and related technologies. In a broader sense, the term characterizes not only a technical development, but also a state-of-mind, a way of thinking, an attitude, and a commitment for networked, global thinking, to improve health care locally, regionally, and worldwide by using information and communication technology” [18]. E-health can thus refer to digital interventions, as well as to technical support for clinicians in the form of electronic health records, electronic referrals and prescriptions, and also to business-related aspects of health-care such as cost-effectiveness of patient-clinician communication, biomedical testing, and prevention measures.

The focus of the present study is on digital interventions as a tool that could improve patient health by offering treatment content in a digital package, where the clinician might more effectively address patient concerns without increasing the burden of work and perhaps simplifying procedures for maximum patient-clinician benefit. The specific aim of the present study was to address the research questions of how clinicians currently support patients to promote a healthy lifestyle, to what extent they are satisfied with current practice and, finally, how they perceive a specified future scenario where digital tools would be available to support patients in changing lifestyle behaviors. The study is thus a qualitative exploration of clinicians’ experiences of healthy lifestyle promotion within primary care, and their perceptions of digital interventions as possible future complements to the personal patient-clinician encounter.

## Methods

### Design

The study had a qualitative design and was exploratory in nature. Data from focus group interviews were evaluated using content analysis [19] with inspiration from phenomenological-hermeneutic methodology, which aims to illuminate the deeper meanings of the phenomenon studied, for example, prejudices and values in relation to the use of digital interventions [20].

### Setting

Data were collected during the spring of 2015. Focus group interviews were conducted at 10 primary health care clinics in three regions in Sweden: the metropolitan areas of Stockholm, with 2.2 million inhabitants, Gothenburg with half a million people, and Linköping/Norrköping region in mid-Sweden with about 300,000 inhabitants. The interviews took place in a secluded setting in each primary care clinic, and lasted between 30 and 65 min. All clinicians at the primary care clinics chose to participate in the interviews during their lunch break or as a replacement for a regularly planned staff meeting. No financial compensation was given, but participants were offered a lunch sandwich or a coffee treat during the interview. The procedure for including interview participants is described below.

### Sampling procedure

We applied purposeful, homogeneous sampling [21], where we sought to recruit clinics with innovation-friendly profiles. For example, in the Linköping/Norrköping region which consists of three geographical areas approximately equal in size, in one area the clinic we chose was a relatively small clinic known for having tested health promotion methods and introducing innovations; the chosen clinic in a second area was large and had previously shown interest for introducing new methods for health promotion; the clinic in the third area was also a larger clinic that had shown interest in testing new health promotion methods. Each clinic that agreed to participate was asked to recruit clinicians from different professions for a focus group interview. The reason for approaching clinicians from different professions was our assumption that clinicians in all primary care professions meet patients that are in need of lifestyle changes, but the focus of the patient-clinician encounter varies by profession; e.g., the physician may focus more on biomedical measures of ill-health, the physical therapist might focus on specific physical exercises to improve specific muscular functions, while the psychologist might focus on motivation and cognitive-behavioral strategies for improving lifestyle behaviors.. We hoped to thus obtain a variety of perspectives on clinicians’ experiences of everyday work and the problems they were encountering in promoting healthy lifestyle. Our intention was also to interview clinicians at primary health care clinics with a particular interest in evidence-based practice, where clinicians might be particularly interested in using digital interventions for healthy lifestyle promotion. Participation was voluntary and clinicians were asked to give about 1 h of their time to the study.

### Data collection

Data were collected using semi-structured focus group interviews, carried out by seven of the authors. Two researchers participated in each of the focus group interviews, where one led the interview and the other had an observer role. Participants were first given information about the aim of the interview, and then filled in questionnaires about the background information, the purpose of which was to clarify the characteristics of the respondents in each focus group; the identity of each questionnaire was connected to the focus group rather than to each individual participant. The interviewer then began asking the interview questions, which the participants answered in an open discussion. When participant responses needed clarification, the interviewer asked follow-up questions so participants could elaborate on what they meant.

Throughout the interview, the interviewers focused on the main phenomenon under investigation: understanding how the participants were currently working with healthy lifestyle behaviors and what that meant, including problems and challenges. The first part of the interview began with a focus on clinicians’ perceptions regarding their ongoing work with healthy lifestyle promotion. The questions asked were as follows: “How do you work with healthy lifestyle behaviors in your primary care clinic today?” “What kind of support do you offer patients with unhealthy lifestyle behaviors?” How do you think your current work with healthy lifestyle behaviors is functioning?; and “Have you considered changing the way you work with promoting healthy lifestyle behaviors?” .

The second part of the interview focused on clinicians’ perceptions of digital interventions for healthy lifestyle promotion, based on any past professional experiences of referral to such interventions, as well as possible visions of future referral to digital interventions for healthy behavior change. The questions concerned how the participants related to any currently used digital intervention (including screening if relevant), as well as thoughts about future implementation of digital tools. Then a scenario was presented in which the participants were asked to imagine a situation where it would be possible to refer a patient to a digital tool for changing healthy lifestyle behaviors, as a complement to the face-to-face consultation. Participants were then asked about advantages and disadvantages they could envision with digital referral. Follow-up questions were asked for clarification purposes.

### Data analysis

All interviews were digitally recorded [22] and transcribed verbatim by a professional transcription service. Data were analyzed in accordance with a phenomenological-hermeneutic model, which encompasses three stages: naïve understanding, structural analysis and comprehensive understanding [20].

- *Stage 1, naïve understanding.* In this stage, each interview was read and listened to several times, in an attempt to gain an initial understanding and sense of the whole. The main purpose of this first stage was to formulate a first interpretation of the meaning of the phenomenon under investigation. A naïve understanding was conceptualized and recorded by two of the authors (EP, KK) and thereafter communicated to the other authors; for a description see under Results.
- *Stage 2, structural analysis.* The main purpose of this stage was to identify various meanings of the phenomenon “understanding how the participants currently work with healthy lifestyle behaviors and what that means, including problems and challenges” and the phenomenon “clinician views on digital interventions for healthy lifestyle promotion, based on any past professional experiences of referral to such interventions as well as possible visions of future referral to digital interventions for healthy behavior change”. When performing the structural analysis all the interviews were reread to identify meaning units covering these two phenomena, where each meaning unit captures the content of what has been said in relation to the phenomenon and consists of one or more sentences or a part of a sentence. The meaning units were then sorted into *condensations* covering the main core of the content in the meaning units. The condensations were then further reduced into *abstractions.* After that the authors (AT, KS, AHB, KK and EP) met to reflect on the abstractions and compare them across all interviews to discern a pattern of themes and sub-themes. *Themes* and *subthemes* were thus extracted based on the abstractions. Table 1 shows examples of how the structural analysis was performed.
- *Stage 3, comprehensive understanding.* In this stage, the findings from the first two stages were further abstracted, deepened and broadened in a comprehensive understanding. This process involved re-reading the meaning units, condensations, abstractions, themes and sub-themes, and was carried out in sequential steps. First, author AT reviewed the material, posing clarifying questions to authors KK and EP when needed. Then, authors AT, KK, EP, KS and AHB met in order to collectively engage in the abstraction, deepening and broadening process of analysis. Finally, the comprehensive understanding was formulated as a result of the authors’ reflection on their pre-understanding of the research area, analysis of the raw data material, in combination with their professional and other life experiences (e.g., as patients). In this study, the comprehensive understanding has been incorporated into the Discussion and offers an interpretation of the results as a whole.

**Table 1:** Example of the structural analysis, from meaning units to themes

Meaning unit	Condensation	Abstraction	Sub-theme	Theme
“So I think we do a good job. Maybe it can be improved. No, but it can maybe be systematized.”	I think we do a good job. Maybe it can be systematized.	We do a good job. It can be systematized.	Striving towards professionalism	Following structured professional practice
“I would like to have more of a system, that is preferably online, a questionnaire before the consultation so that gets done, so I don’t have to ask or forget or so it gets done, definitely, so I think it works like that with one, yes so that’s the way it can work.”	I would like to have more of a system, preferably online, a questionnaire before the consultation so that gets done.	I want more of a system, an online questionnaire before consultation.	Embracing the future with critical optimism	Following structured professional practice
“But we cannot really offer as much in the current situation as we might want to, a bit more prevention or, there aren’t those possibilities.”	We cannot offer as much in the current situation as we might want to.	We want to offer more – but cannot.	Being in an unmanageable situation	Deficiency in professional practice
“And sometimes I also bring up the other lifestyle behaviors but it’s not always at all, but rather when it feels relevant or appropriate.”	Sometimes I also bring up other lifestyle behaviors but it’s not always but rather when relevant or appropriate	Other lifestyle behaviors brought up only when relevant or appropriate.	Following one’s perception	Deficiency in professional practice

In summary, the analytic procedures were conducted by five of the authors (AHB, KK, EP, KS, AT), who were involved throughout the process of analysis. All participated in formulating new questions to the text, where the analysis was mainly conducted by EP and KK under AT’s supervision. Preliminary results were reflected upon in repeated meetings between all five authors, and the final results were discussed at a meeting with three additional authors (PB, CL, UM). Finally, results were circulated for comments to the remaining authors (EH, FS, IG).

## Results

In this section, we describe the participants, followed by the naïve understanding of the results, and the complete structural analysis of the interview transcripts.

### Participants

Each of the 10 focus group interviews included 3–7 clinicians, with a total of 46 participants who were 85% women with a mean age of 54 years. The following clinical professions were represented: physicians, nurses, physiotherapists, psychologists, social workers, nutritionist, occupational therapist, nursing assistant and medical secretary. About two thirds of the participants reported that they worked fulltime and the rest part-time. Most reported that they discussed lifestyle behaviors with their patients at least five times each week. All participants reported that they had positive or mixed experiences of working with healthy lifestyle promotion. None of the participants reported that their experiences were mostly negative. Due to human error, data on the participants were available for only 9 of the 10 clinics. Table 2 shows an overview of participant characteristics.

**Table 2:** Focus group participant characteristics from 9 primary care clinics (*n* = 41)^a,b^

	Count (n)	Proportion (%)
Women	35	85.4
Mean age (SD)	50.5 (10.0)	
Profession		
Physician	10	24.3
District nurse	5	12.2
Registered nurse	12	29.3
Psychologist	5	12.2
Social worker	2	4.9
Physiotherapist	4	9.8
Nutritionist	1	2.4
Registered nurse in specialist training	1	2.4
Medical secretary	1	2.4
Full-time employee (100% of fulltime)	31	75.1
Part-time employee (75–90% part-time)	10	24.4
General practice clinic	20	55.6
General practice clinic with specialist competence	13	36.1
Individual patient visits per week (mean; SD)	30.7 (20.0)	
How large a proportion of your patients do you ask about lifestyle behaviors? (mean; SD)		71.6 (25.8)
How many times do you bring up lifestyle behaviors with your patients during a normal week’s work?		
< 5 times	6	19.4
5 times or more	25	80.6
Your experience of working with healthy lifestyle promotion		
Mostly positive	21	55.3
Mixed experiences	17	44.7
Mostly negative	0	0

^a^Data were available for 9 of the 10 clinics where focus group interviews were conducted

^b^Participants responded to questionnaires handed out at the beginning of the interview. Where “n” differs from 41, data are missing

### Naïve understanding

Working with lifestyle behaviors, with or without digital intervention, was perceived by participants as a positive challenge, requiring them to adopt new methods and establish structure. Working with healthy lifestyle promotion via digital tools was perceived as being professionally competent and skilled, and using evidence-based practice to achieve the patient’s goals. Working with digital tools was also perceived as a possibility to develop as a professional and to gather momentum for improving practice by means of the digital structure. Working with digital intervention was perceived as positive as a complement to ordinary treatment but the idea of introducing digital interventions was also tied to a feeling of fear: a fear of decreasing face-to-face time with the patient. Furthermore, the possible use of digital interventions was perceived as a change in practice that would evoke an increased struggle with high workload and limited time and organizational resources. Such a new complementary tool could lead to the clinician losing control and sometimes abandoning established routines for the clinical situation.

### Structural analysis

Findings from the structural analysis are presented within two themes that encompass two sub-themes each (see the last two columns in Table 2 for the sub-themes and themes).

#### Following structured professional practice

The *following structured professional practice* theme was illustrated by the sub-themes *striving towards professionalism* and *embracing the future with critical optimism.*

*Striving towards professionalism*, which for our participants meant increasing the quality of their professional competence by working in a standardized fashion, including the possibility of monitoring statistics on a group and individual patient level, as well as working in a similar way regardless of healthcare clinic affiliation.

“…*A standard in the entire county, where one would be able to…extract follow-up statistics; that is, how we [should] work with these [behaviors], diet, physical activity, alcohol and tobacco use.”*

Striving towards professionalism was also viewed as clinicians working with evidence-based practice, as well as exploring the best way to promote health together with the patient.

“ *I have participated in training for Motivational Interviewing (MI) [note: perceived as evidence-based practice]…and I find this is very useful when I talk about health with my patients….”*

The theme of striving towards professionalism also included using a patient-centered approach with personal tailoring, such that healthcare is adapted to each individual patient’s needs.

“*I try…together with the patient…to find the most appropriate form of activity s/he…thinks would be good to use.”*

*“Then you have to adapt [the method] because the patient does not only have chronic obstructive pulmonary disease (COPD), but also…this and that and this too…so you can provide care according today’s evidence level [for each of the problems].”*

Furthermore, striving towards professionalism was seen as sharing new views and opportunities within clinicians’ inter-professional teamwork, and working towards an integrated, holistic view of the patient.

*“We have been trained in different ways but we need the whole [picture] around the individual, and that is working as a team.”*

The second sub-theme illustrating the theme of *Following structured professional practice* was *Embracing the future with critical optimism*, which meant a willingness to develop professionally: being full of expectations for the future and welcoming changes in the current way of working.

*“For us this is something we have demanded for many years, this specific availability to use digital technology in some way.”*

*Embracing the future with critical optimism* also included concern about the consequences of integrating digital tools into primary care, in terms of the importance of personal interaction between patient and provider. Specifically, the challenge concerns how digitalization can be implemented as a complement, but not as a replacement for the encounter between clinicians and patients.

*“And if it were clear that [digital tools] are a complement to the care that the person receives…because I think the personal encounter is also important to retain.”*

Embracing the future with critical optimism also meant expectations about quality assurance in terms of efficient follow-up of patient outcomes and reliance on the evidence base for digital tools. Clinicians hoped for access to regular feedback on patient outcomes, both at group and individual levels.

*“I would very much like to have results on a group level…this kind of feedback would help me feel confident in referring [patients] further if I saw that they were helped.”*

Furthermore, the embracing the future with critical optimism sub-theme also involved personal interaction and digitalization, where the latter was experienced as positive when it was a complement to the encounter between clinicians and patients.

*“Yes, although I think it [digitalization] would unburden us a great deal since we have such a darn huge influx - and suppose one could have alcohol treatment online instead and have two sessions, one before and one after, instead of maybe five [personal sessions].”*

### Deficiency in professional practice

The second theme, *deficiency in professional practice*, was illustrated by the sub-themes *Being in an unmanageable situation* and *Following one’s own perception.*

*Being in an unmanageable situation* meant not being able to influence one’s work situation. It was experienced as being limited to working with the patient’s immediate agenda and sacrificing ambitions of working with healthy lifestyle promotion.

*“As things are at the moment, we cannot offer as much, really, as we would like to: [we would like to work a bit more preventively, but]…these possibilities are not available. The influx of new patients with…emergency problems is so intense.”*

Being in an unmanageable situation was expressed as not being able to do what’s best for the patient. It was experienced as lacking time and resources and being forced to ignore what is best for the patient’s health.

*“But we notice of course that [for] those we frequently follow up things with…we get a better result. But one has to let go of that because we don’t have those resources…those one can follow up very frequently get better results most often, that’s [simply] the way it is.”*

Being in an unmanageable situation also involved difficulties in adapting to challenges related to new technology and organizational structures, such as implementation of new guidelines or routines that turned out to be obstacles instead of facilitating care. This was made clear by clinicians who expressed hesitancy in relation to new routines when these required more commitment from them.

*“As as long as [new routines] do not involve a lot of administration that does not generate revenue [for the clinic]. For then I think we have a giant obstacle, then we will push it away from us, I think. But on the other hand, if [new routines] unburden some things and leave time for other tasks, then [they] could lead to the opposite effect.”*

The second sub-theme illuminating *Deficiency in professional practice* was *Following one’s own perception*. Following one’s own perception meant that doubts in relation to new methods or guidelines led to reflection on which of these actually might be effective or useful. Clinicians expressed their experience that new routines reduced the possibility of working with lifestyle issues.

*“Yes, one can [wonder] “whose idea was that?” but the way [things are], previously we had preventive information within maternal care, for example for pregnant women, but we are not allowed to [now], We can thus have groups for pregnant women with pregnancy-related symptoms but we are not allowed to use these for prevention and really, it’s also like that for lifestyle promotion. It’s rather absurd, we want to encounter patients who want help anyway, so in some way we resolve it, but [prevention] is not part of our agenda. ”*

Following one’s own perception involved treating patients according to the clinicians’ own preconceptions and was experienced as using a gut feeling in the interaction with the patient.

*“If someone comes in and weighs 110 kilograms and it smells of sweat in the whole room and it stinks of smoke, and [s/he] has a blood pressure of 200/100, well then one maybe thinks that perhaps lifestyle behaviors are on the menu, right.”*

Following one’s own perception also included articulating that one’s own prejudices could determine clinical practice. It was experienced as choosing which patient was most worthwhile to work with.

*“Yes, it’s clear, we want to reach the younger ones so to speak, those who still have possibilities of changing.”*

Furthermore, following one’s own perception meant freely choosing how to treat the patient. This was visualized as the clinician following his/her gut feeling and could be described by participants as the clinician deviating from clinic routines.

*“No, but it is like this, our problem is to a certain extent that physical activity is part of our work so we ask almost all the patients about this, but not according to the questionnaire that is in the computer.”*

Following one’s own perception could also involve working unsystematically, so that the work was experienced as being carried out in an unstructured manner. This was made clear by the random nature of questions being asked in patient encounters.

*“I can ask about depression…stress too, if one eats more than usual,[or] less; it is arbitrary, like when I ask about diet – it is not a standard routine.”*

## Discussion

The purpose of this study was to elucidate the perceptions of clinicians in primary care on healthy lifestyle promotion, with or without digital intervention. The results showed that the clinicians in this study were occupied with a quest to find the best way to promote patient health, in structured professional practice. Their strategy frequently involved working with evidence-based practice, as shown in the sub-theme *striving towards professionalism*, and their ambitions included *embracing the future with critical optimism*, looking ahead towards the possibility of enhancing and facilitating healthcare by using digital tools. At the same time, the clinicians’ quest was also characterized by deficient professional practice, which was expressed as *being in an unmanageable situation* and then resorting to *following one’s own perception* on how to engage the patient in the consultation. Clinicians seemed to be trapped between their own ambitions to practice effectively and the uncertainty they were faced with when dealing with time- and resource-related barriers to structured practice and navigating in clinical situations where they experienced a need to follow their gut feeling.

Our *comprehensive understanding* of the results, presented here, is our interpretation of the whole corpus of results where the themes have been merged, yielding an image of *the clinician alternating between structured and deficient professional practice*. The rhythm of this alternation was unpredictable and complex. The clinicians’ experience was that practice was often associated with unmanageable situations, where their ambitions reached beyond actual practice. Asking questions about lifestyle behaviors could lead to losing control of time, shifting the clinician into the “gut feeling” mode more characteristic of deficient professional practice. In some consultations, the ambition to work in a structured manner could be carried out, where the clinician could actually address lifestyle behaviors in a relevant and appropriate manner. The clinicians thus experienced occasional successful allegiance to the structured practice they were generally striving towards, and then shifted into deficient or sub-optimal practice when time and other organizational demands led to their experiencing an unmanageable situation regarding addressing patients’ lifestyle behaviors.

We now turn to discussing our results in relation to relevant research literature. The clinicians in the present study alternated between structured and deficient professional practice. In their citations a sense of discomfort and frustration is evident, but it is not clear to what extent their working conditions are affected overall. Negative working conditions can be related to increased emotional exhaustion and burnout among staff if work demands are high and the possibility of controlling one’s work situation is low [23], as well as when one’s efforts are not perceived as generating sufficient reward [24]. The question of the quality of working conditions and reducing burnout was addressed in a randomized controlled study that found that burnout and dissatisfaction were reduced and staff retention improved by addressing communication and workflow as well as initiating targeted quality improvement projects [25]. Our findings show a complex movement between having the intention and willingness to work according to evidence-based practice but at the same time not having the ability or the readiness to work this way, partly due to personal reservations concerning the patient-clinician relationship, and partly due to organizational challenges concerning both the clinician’s workload and difficulties related to the successful introduction of new routines and technology.

Regarding the application of evidence-based practice, a 2008 survey of nurses showed that their belief in the value of using evidence-based practice is an important means for improving the quality and results of the care given [26]. However, clinicians’ attitudes to working with evidence-based practice have been shown to be more positive than their actual knowledge and skills concerning evidence-based practices [27], as well as the extent to which implementation of evidence-based practice can be incorporated into daily practice [28]. In the present study the sub-theme of *being in an unmanageable situation* suggested that clinicians had difficulties incorporating new technology and practices such as implementation of new guidelines or routines. An earlier qualitative study of physician’s perceptions showed that while benefits of evidence-based practice were perceived, it was crucial to continue to acknowledge the importance of clinical expertise and patient preference [29]. This was shown in the present study in the theme *striving towards professionalism* when the clinicians expressed their perceived need to use a patient-centered healthcare approach with personal tailoring to meet the patient’s needs.

Evidence-based practice could potentially include digital tools in the future, and it emerged in the present study that *embracing the future with critical optimism* meant that clinicians are processing the idea of possibly integrating digital tools into primary care. The clinicians in our study were asked to discuss the future use of internet-based interventions as a complement to traditional face-to-face care. The clinicians expressed a positive attitude, looking forward to the opportunity to work with digital interventions in the future, but expressed that it was important that such tools not replace personal interaction altogether, a finding also included in the sub-theme of *embracing the future with critical optimism.*

It is not surprising that our respondents perceived future introduction of digital interventions into the workplace with some trepidation. A qualitative study analyzing the question of why e-health initiatives are so difficult to implement [30] found that Normalization Process Theory (NPT) helps to explain how optimal “normalization” of e-health initiatives occurs. It is vital to generate positive effects in interactions between patient-clinician dyads as well as inter-professional groups. According to NPT, successful e-health implementation is more likely when the e-health initiative is well aligned with existing staff skills as well as with the goals of the healthcare organization. Despite the difficulties inherent in introducing digital interventions, research suggests that such tools could be part of a general overall improvement in the efficiency and effectiveness of primary health care. A recent systematic review of digital and computer-based interventions for alcohol abuse in primary care argued that digital and computer interventions could help health care providers overcome current obstacles – “a lack of time, training and resources and concerns about adverse effects on rapport with patients” – in providing effective face-to-face interventions for problematic alcohol use among patients in primary care [16]. Although relatively few studies have evaluated the effects of digital interventions in primary care, the review authors conclude that additional studies in this area could potentially show cost-effectiveness and reduce provider burden for digital interventions. An additional challenge, however, is maintaining ethical standards of care; e.g., ensuring that the digital tools function as meaningful and useful complements to the standard face-to-face interaction between patient and clinician, not as cheap replacements for just some of the components of quality care [31]. The prospect of introducing digital tools into primary care is clearly challenging, given the complexity of clinician concerns as well as clinicians’ expectations and hopes for technological solutions. Within the sub-theme *embracing the future with critical optimism,* clinicians in the present study expressed concern about maintaining a personal relationship with the patient when using digital tools, as well as concern about the quality assurance of monitoring treatment results, hoping that they might obtain regular feedback on individual and group levels.

Clinicians in the present study who experienced *being in an unmanageable situation* described difficulties in changing the way health care providers work today and in implementing evidence-based practice when time and resources are perceived as lacking. In the qualitative study of physicians cited earlier, the most common impediment to the exercise of evidence-based practice was having enough time in relation to an extremely high workload [29]. In the present study, this was associated with *following one’s own perception*, where the clinician freely chose how to treat patients and then deviated from clinic routines. This experience was also reflected in the physician study, where all participants reported that they had experienced conflict in their efforts to practice evidence-based practice. After physicians reviewed the available scientific evidence, the study showed that it was not unusual for them to practice in violation of the evidence because they felt that following the evidence would not apply to their specific clinical context [29]. The current study found a similar phenomenon, where the clinician often acted according to his or her own preconceptions, using their gut instinct when meeting patients but experiencing conflict since clinicians did not perceive themselves as following evidence-based practice in some of these cases. Gut feelings can, however, have a clear diagnostic role, as shown in a focus study with physicians in general practice, who were all familiar with the phenomenon and indeed relied on it, both for reassurance regarding diagnosis and choice treatment as well as for alerting them when something seemed “wrong” without objective evidence [32].

This study was based on focus group interviews with a variety of clinicians from the primary care sector. To maximize validity and reliability in our results, we followed quality criteria for qualitative studies such as credibility, transferability/generalizability and dependability [21]. *Credibility* was sustained in this study by interviewing a varied sample of clinicians from different professions, at different health clinics, in different parts of Sweden. The authors analyzing the data and performing most of the interviews have a background as clinicians, which enhanced researcher credibility. To achieve in-depth interviews, each interviewer adopted a naive stance in relation to the participants’ responses and used follow-up questions to minimize unfounded assumptions regarding the understanding of participant answers. To validate *transferability/generalizability*, the preliminary results of the study were presented for the other clinical researchers in our group. These individuals were not intimately involved with the data analysis and interpretation, and expressed a sense of recognition from their clinical and research experiences in primary care, in relation to the data interpretation. *Dependability* refers to the stability of data over time, from a technical and analytical perspective. The interviews were transcribed verbatim directly after recording them. During the analysis, subjective interpretations were minimized by ensuring that the authors independently analyzed the transcribed text and only worked together afterwards to compare and discuss the findings. Nonetheless, the interpretations made in the study are only some of several possibilities. The findings may not be said to represent an objective truth but instead are interpretations of the reality experienced and presented by the participants. Our findings may thus not be widely generalizable. Finally, although the sample was varied in many aspects, its size was small, a circumstance that may limit its representativity.

## Conclusions

In conclusion, this study contributed findings based on a content and phenomenological-hermeneutic analysis of focus group interviews with inter-professional clinicians from the primary care context, where the focus was clinicians’ current and future envisioned work with healthy lifestyle promotion. Our discovery that clinicians perceived themselves as alternating between structured and deficient professional practice illuminates some of the complexities involved in introducing e-health initiatives to provide digital tools as complements to clinicians’ efforts to address lifestyle behaviors in the primary care encounter.

In terms of specific clinical implications, we believe our findings can be of help to decision- and policy-makers planning the introduction of such digital tools, in their efforts to increase evidence-based practice and lighten the burden of clinicians in primary care. The findings show a need to maintain a balanced view on digital interventions, explicitly stating their role as *complements* rather than replacements of face-to-face encounters. Introducing digital interventions for healthy lifestyle promotions in clinical practice should thus be done in a way that allows for personalized patient encounters alongside standardized and evidence-based practice. Our overriding hope is that this study will contribute to maintaining meaningfulness in the patient-clinician encounter, when digital tools are added to facilitate patient behavior change of unhealthy lifestyle behaviors.

## Additional file

Additional file 1: Participant consent form. (PDF 57 kb)

## Abbreviations

COPD: Chronic obstructive pulmonary disease
MI: Motivational interviewing
NPT: Normalization process theory
PPG: Grant for patient-focused research and development in Primary Care, Psychiatry and Geriatrics
