# Supplementary material for: Clinician experiences of healthy lifestyle promotion and perceptions of digital interventions as complementary tools for lifestyle behavior change in primary care
Source: BMC Fam Pract. 2018 Aug 21;19:139. doi: 10.1186/s12875-018-0829-z (PMC6103870; doi:10.1186/s12875-018-0829-z)
Supplement: Supplementary file 1 — Participant consent form. (PDF 57 kb) [file 12875_2018_829_MOESM1_ESM.pdf]

Medgivandebrev för deltagare i studien

”Fokusgrupp om implementering av digitala hälsointerventioner i primärvården”

Jag har fått tillräcklig information om studien:

Ja ☐ ☐ Nej ☐☐

Jag har fått information om syftet med denna intervju/fokusgrupp och hur den kommer att genomföras:

Ja ☐☐ Nej ☐

Jag har fått information om att jag när som helst under studiens genomförande kan avbryta min medverkan, utan att behöva ge någon förklaring och utan några som helst konsekvenser för min del:

Ja ☐☐ Nej ☐ ☐

Jag ger mitt medgivande till att det jag säger under intervjun/fokusgruppen får användas som data i denna studie:

Ja ☐☐ Nej ☐ ☐

Jag ger mitt medgivande till att delta i denna studie:

Deltagarens signatur:____

Namnförtydligande, ort och datum:____

Fylls i av ansvarig forskare:
Jag anser att personen/erna som signerat detta medgivande förstår vad studien innebär och frivilligt deltar i studien:

Forskarens signatur:____

Ort och datum: ____
